# Oxidative Stress and* Salvia miltiorrhiza* in Aging-Associated Cardiovascular Diseases

**DOI:** 10.1155/2016/4797102

**Published:** 2016-10-11

**Authors:** Cheng-Chieh Chang, Yu-Chun Chang, Wen-Long Hu, Yu-Chiang Hung

**Affiliations:** ^1^Department of Chinese Medicine, Kaohsiung Chang Gung Memorial Hospital and Chang Gung University College of Medicine, Kaohsiung, Taiwan; ^2^Department of Applied Cosmetology, Kao Yuan University, Kaohsiung, Taiwan; ^3^Kaohsiung Medical University, College of Medicine, Kaohsiung, Taiwan; ^4^Fooyin University College of Nursing, Kaohsiung, Taiwan; ^5^School of Chinese Medicine for Post Baccalaureate, I-Shou University, Kaohsiung, Taiwan

## Abstract

Aging-associated cardiovascular diseases (CVDs) have some risk factors that are closely related to oxidative stress.* Salvia miltiorrhiza* (SM) has been used commonly to treat CVDs for hundreds of years in the Chinese community. We aimed to explore the effects of SM on oxidative stress in aging-associated CVDs. Through literature searches using Medicine, PubMed, EMBASE, Cochrane library, CINAHL, and Scopus databases, we found that SM not only possesses antioxidant, antiapoptotic, and anti-inflammatory effects but also exerts angiogenic and cardioprotective activities. SM may reduce the production of reactive oxygen species by inhibiting oxidases, reducing the production of superoxide, inhibiting the oxidative modification of low-density lipoproteins, and ameliorating mitochondrial oxidative stress. SM also increases the activities of catalase, manganese superoxide dismutase, glutathione peroxidase, and coupled endothelial nitric oxide synthase. In addition, SM reduces the impact of ischemia/reperfusion injury, prevents cardiac fibrosis after myocardial infarction, preserves cardiac function in coronary disease, maintains the integrity of the blood-brain barrier, and promotes self-renewal and proliferation of neural stem/progenitor cells in stroke. However, future clinical well-designed and randomized control trials will be necessary to confirm the efficacy of SM in aging-associated CVDs.

## 1. Introduction

Cardiovascular diseases (CVDs) are a group of disorders related to the heart or blood vessels. Major CVDs include stroke, ischemic heart disease, cardiomyopathy, rheumatic heart disease, hypertensive heart disease, endocarditis, atrial fibrillation, aortic aneurysm, and peripheral arterial disease [1]. Global life expectancy increased from 65.3 years in 1990 to 71.5 years in 2013. At the same time, the numbers of deaths from noncommunicable diseases increased steadily [2]. CVDs are the leading form of noncommunicable diseases [2]. In 2012 and 2013, 17.3 million deaths worldwide resulted from CVDs [3]. Among these deaths, coronary artery disease and stroke contributed most to the total global burden of CVDs [1]. It is estimated that 90% of CVDs are preventable [4]. The Framingham and World Health Organization MONICA studies found several risk factors for CVDs (e.g., age, smoking, physical inactivity, unhealthy diet, obesity, family history, hypertension, diabetes mellitus, and hyperlipidemia) [5–10]. Some of these risk factors are immutable; however, many important risk factors are modifiable. When relevant risk factors decrease, the incidence and mortality of CVDs improved.

Most CVD risk factors are related to oxidative stress. Reactive oxygen species (ROS) are the main cause of oxidative stress and are highly reactive with proteins, lipids, and DNA, damaging these cellular components [11]. Under normal conditions, the production of ROS during aerobic metabolism and the scavenging of ROS by tissue antioxidant systems are in balance [12]. This balance is shifted in favor of oxidative stress in the presence of cardiovascular risk factors [5, 13, 14].

**Figure 1:**
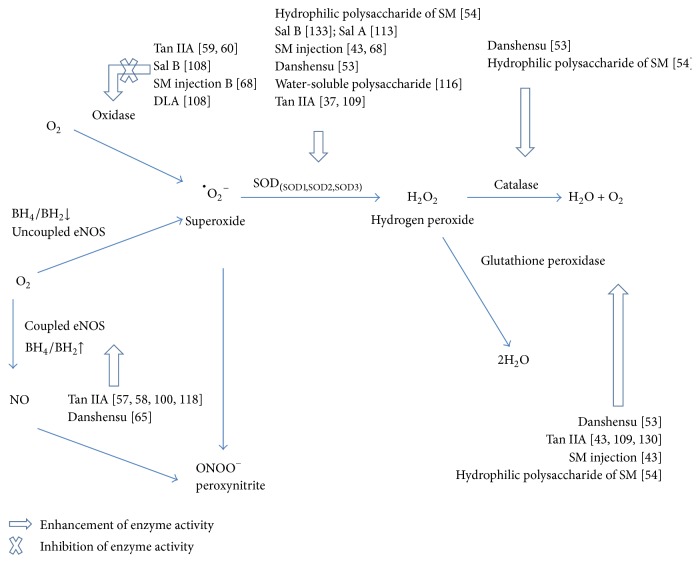
Vascular reactive oxygen species production. Oxidases convert oxygen to superoxide, which is then dismutated to H_2_O_2_ by superoxide dismutase (SOD). H_2_O_2_ can be converted to H_2_O by catalase or glutathione peroxidase. In addition, coupled endothelial NO synthase (eNOS) catalyzes the formation of nitric oxide (NO). When tetrahydrobiopterin (BH4) generation is reduced, the uncoupled eNOS produces superoxide instead of NO. The superoxide can react rapidly with NO to form peroxynitrite (ONOO^−^), a powerful oxidant and nitrating agent. Reference numbers are inside the parentheses. DLA: 3,4-dihydroxyphenyl lactic acid; SM:* Salvia miltiorrhiza*; Sal A: salvianolic acid A; Sal B: salvianolic acid B; Tan IIA: tanshinone IIA.

The most important forms of ROS are nitric oxide (NO), superoxide, hydrogen peroxide, and peroxynitrite (Figure 1). NO is produced in normal physiologic conditions from L-arginine by coupled endothelial nitric oxide synthase (eNOS) that is activated via protein kinase A- or Akt-dependent phosphorylation [15]. NO is a crucial mediator of blood vessel homeostasis by inhibiting vascular smooth muscle contraction and growth, platelet aggregation, and leukocyte adhesion to the endothelium. Under some circumstances, such as hypertension, hyperglycemia, and hypercholesterolemia, eNOS becomes uncoupled and superoxide is synthesized rather than NO [16–20]. When normal NO production is impaired, CVDs may occur [21].

In aged vessels, endothelial dysfunction occurs owing to eNOS uncoupling by reducing the tetrahydrobiopterin to dihydrobiopterin ratio [22]. Superoxide is the product of a univalent reduction of oxygen by various oxidases [23]. The important oxidases include xanthine oxidase, uncoupled NO synthases, cytochrome P450 enzymes, and mitochondrial and NADPH oxidases [24–26]. Superoxide is toxic and possesses three main effects that contribute to the pathogenesis of CVDs [27]: (1) rapid inactivation and reaction with NO to form the more highly reactive peroxynitrite; (2) mediation of aberrant redox signaling in the vasculature to induce alterations of vascular function, and (3) direct oxidative damage to cell components [28].

Peroxynitrite is a powerful oxidant and nitrating agent. Peroxynitrite can damage a wide array of cellular molecules, including carbonate, proteins, low-density lipoproteins (LDL), and DNA to form nitration and nitrosation products that are involved in the pathogenesis of cardiovascular complications [29, 30].

The superoxide dismutases (SODs) are the major antioxidant enzymes that degrade superoxide. There are three isoforms of SOD in mammals: cytoplasmic Cu/ZnSOD (SOD1), mitochondrial MnSOD (SOD2), and extracellular Cu/ZnSOD (SOD3) [31]. All forms of SOD rapidly dismutate superoxide to the more stable ROS, hydrogen peroxide, that is then converted to water and oxygen by either catalase or glutathione peroxidase [32].

*Salvia miltiorrhiza* (SM) belongs to the family of Labiatae and its dried root, referred to as “Danshen” in traditional Chinese medicine, has been commonly used for hundreds of years in the treatment of CVDs [33]. Our previous population-based studies demonstrated that SM is the most common herbal drug used to treat ischemic heart disease [34] and ischemic stroke [35]. In traditional Chinese medicine, Danshen is regarded as an important herb for “activating circulation and dispersing stasis or sludging of blood.” SM exhibits strong antioxidant activity by scavenging ROS [36]. SM also modulates endothelial cell permeability, inhibits platelet aggregation [37], and protects human umbilical vein endothelial cells against homocysteine-induced endothelial dysfunction [38] or vascular smooth muscle cells proliferation [39].

**Figure 2:**
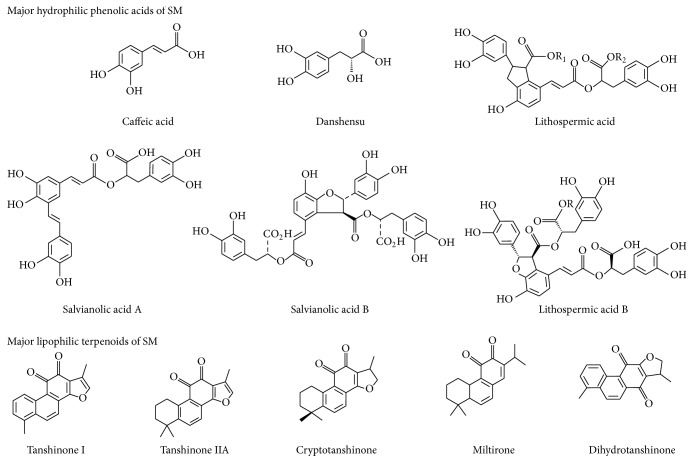
The chemical structure of major hydrophilic phenolic acids and lipophilic terpenoids of* Salvia miltiorrhiza*.

There are many active constituents found in alcohol and water extracts of SM (Figure 2). At least 49 diterpenoid quinones, more than 36 hydrophilic phenolic acids, and 23 essential oil constituents have been isolated and identified from SM [40]. Among the chemical components, tanshinone IIA (Tan IIA) and salvianolic acid B (Sal B) are usually chosen as markers to assess the quality of SM [41].

Terpenoids are the most important lipophilic components of SM. Most diterpenoids are present as tanshinones and their analogs, which are a class of abietane diterpene compounds found exclusively in the Salvia genus [42]. Tanshinone I, Tan IIA, and cryptotanshinone are the major constituents of the tanshinones and are studied mainly for their biological activity [43]. SM also contains some triterpenoids [44]. These terpenoids possess a wide range of biological activities including antioxidant [45], antibacterial [46], anti-inflammatory [47], antiatherogenic [36], neuroprotective [48], antitumor [49], and antidiabetic [50] effects.

Phenolic acids are the main type of hydrophilic component from SM. Most of the phenolic acids in SM are condensation derivatives of caffeic acid in different linkage forms and numbers [51]. The most prominent effects of the phenolic acids in SM are antioxidant, anticoagulant, and antithrombotic activities and protection of cells from ischemia-reperfusion (I/R) injury [33, 52, 53]. Moreover, over the past few years, there are several studies showing that the polysaccharides extracted from SM also have antioxidant and antitumor activities [54, 55].

Unfavorable changes in CVD risk factors are seen in aging individuals and will likely be reflected in worsening morbidity and mortality [56]. Thus, the minimization of risk factors is a key point for decreasing the incidence of aging-related CVD.

Many researchers have demonstrated that SM can reduce the impact of CVD risk factors and the injury caused by oxidative stress. This article provides an overview of SM in terms of its ability to reduce oxidative stress-induced injury and aging-related risk factors associated with CVD. Studies of the effects of SM on the two most frequent CVDs, coronary artery disease and stroke, are also reviewed.

## 2. Materials and Methods

The current review focuses on the role of oxidative stress and SM (Danshen) in aging-associated CVDs. Literature searches were done using the Medicine, PubMed, EMBASE, Cochrane library, CINAHL, and Scopus databases, and the contents of the identified articles were summarized.

## 3. Results and Discussion

### 3.1. Hypertension

Hypertension is the most readily modifiable risk factor for CVDs [57]. Oxidative stress, an aberrant vascular redox system, and endothelial dysfunction can contribute to hypertension [13, 28]. In traditional Chinese medicine, Danshen is the most frequently prescribed single herb for hypertension [58]. Tan IIA has a vasodilatory effect through restoring eNOS coupling by increasing the ratio of tetrahydrobiopterin to dihydrobiopterin and reducing the production of superoxide by inhibiting the expression of NOX4, a member of the NADPH oxidase family [59, 60]. In addition to reducing ROS, Tan IIA protects against endothelial cell damage by decreasing the Bax/Bcl-2 ratio and inhibiting caspase-3 activation [61]. The water extract of SM that contains lithospermic acid B (also named tanshinoate B) and Sal B exhibits an antihypertensive effect through the inhibition of angiotensin-converting enzyme or the renin angiotensin system [62–65]. Sal B and danshensu, the major hydrophilic constituents of SM, can regulate vascular tone and reduce blood pressure by activating of the calcium-activated big potassium (BK_CA_) channel [66, 67]. In addition, SM injection can decrease plasma levels of endothelin-1 and thromboxane B2 [68].

### 3.2. Smoking

Cigarette smoking is an important and reversible risk factor for CVDs but is ranked lower than hypertension because of the widespread implementation of smoke-free legislation [69]. However, in some countries, smoking remains the third leading risk factor for CVDs, behind dietary risks and hypertension. Smokers return to the risk level of never-smokers after cessation of smoking for at least 10 years [69].

Smoking may enhance oxidative stress not only through the production of ROS but also through weakening of the antioxidant defense systems [70]. Smoking-associated CVDs include abdominal aortic aneurysm, peripheral artery disease, unheralded coronary death, and subarachnoid hemorrhage [71]. SalA attenuates the formation of aortic aneurysms in apolipoprotein E-deficient mice by selectively inhibiting matrix metalloproteinase-9 (MMP-9) to maintain the integrity of blood vessels [72]. A crude extract of SM dilates isolated rat femoral arteries by opening tetraethylammonium-sensitive potassium channels in smooth muscle cells [73] and produces a vasorelaxant effect in the rat knee joint through the release of calcitonin gene-related peptide and the endothelium-derived relaxant factors NO and prostaglandins [74]. One recent study revealed that the injection of Danshen root could suppress cigarette smoking-induced lung inflammation by decreasing the levels of interleukin- (IL-) 8, IL-6, and tumor necrosis factor- (TNF-) *α* in Sprague-Dawley rats [75].

### 3.3. Hyperglycemia

Hyperglycemia and diabetes mellitus are strong, significant, and independent risk factors for CVDs [76, 77]. Hyperglycemia induces oxidative stress in diabetic patients, and the overproduction of ROS contributes to the development of CVDs [78, 79]. Peroxynitrite plays an important role in the pathogenesis of diabetic CVD complications through oxidative and nitrosative stress [29]. In the presence of hyperglycemia, vascular remodeling is augmented by uncoupled eNOS [80], increases in endothelial superoxide levels that inhibit vascular smooth muscle Na-K-ATPase activity [81], and downregulation of transient receptor potential cation channel subfamily V member 4 that regulates vascular function [82]. The hydrophilic extract of SM clearly ameliorates oxidative stress stimulated by hyperglycemia in diabetic patients with coronary heart disease [83]. The induction of vascular endothelial growth factor (VEGF) expression by high glucose levels is also reversed by the SM hydrophilic extract through amelioration of mitochondrial oxidative stress [84].

Sal B is the main bioactive component in the SM hydrophilic extract [85]. The tanshinones are insulin sensitizers that enhance the activity of insulin on tyrosine phosphorylation through the activation of Akt and extracellular signal-regulated kinase (ERK)1/2 and by glycogen synthase kinase (GSK)3*β* and glucose transporter (GLUT)4 translocation [86]. Furthermore, the hydrophilic polysaccharide of SM protects against the development of type 2 diabetes by attenuating insulin resistance through increases in the activities of catalase, MnSOD, and glutathione peroxidase in rats [54].

### 3.4. Hyperlipidemia

Hyperlipidemia is the most important risk factor for atherosclerosis and a major cause of CVDs [87, 88]. Increased transcytosis of lipoproteins is the initial event in atherogenesis. ROS generated by activated inflammatory cells and the production of oxidized lipoproteins are key points for atherosclerotic plaque erosion and rupture [89]. We showed that Tan IIA exhibits a strong antiatherosclerotic effect associated with reduced vascular cell adhesion molecule- (VCAM-) 1, intercellular adhesion molecule- (ICAM-) 1, and CX3CL1 expression through inhibition of the NF-*κ*B signaling pathway in human vascular endothelial cells [90]. Sal B inhibits LDL oxidation and neointimal hyperplasia in endothelium-denuded hypercholesterolemic rabbits through inhibition of ROS production [91]. Magnesium tanshinoate B, an important aqueous component of SM, can also inhibit oxidative modification of LDLs, prevent the uptake of LDLs by macrophages [92], and protect endothelial cells against oxidized lipoprotein-induced apoptosis [93].

### 3.5. Overweight and Obesity

Obesity has become a global epidemic. The 2013 National Health and Nutrition Examination Survey (NHANES) guidelines recommended 64.5% of American adults for weight loss treatment [94]. Obesity is an independent risk factor for CVDs [95]. Adipose tissue is a significant source of TNF-*α*, IL-6, resistin, leptin, angiotensinogen, and adiponectin [96]. The production of these proinflammatory cytokines may contribute to the low-level systemic inflammation seen in obesity-associated chronic pathologies [97].

Endothelial dysfunction is present in obese individuals due to decreased NO and increased oxidative stress [98]. Cryptotanshinone inhibits phosphorylation of STAT3 during early adipogenesis and then downregulates the expression of the early transcription factors C/EBP*β* and PPAR*γ* to suppress preadipocyte differentiation [99]. Sal B can also suppress the expression of PPAR*γ* and C/EBP*α* and increase the expression of GATA binding proteins 2 and 3 to prevent the differentiation of preadipocytes and weight gain in obese mice [100]. There is also a study in a rodent model demonstrating that a Chinese herbal extract (SK0506) containing SM possesses a favorable impact on the metabolic syndrome through suppression of visceral fat accumulation and regulation of lipid metabolism [101].

### 3.6. Coronary Artery and Ischemic Heart Diseases

#### 3.6.1. Angina

Angina pectoris and coronary artery spasm are the most common coronary artery diseases. Tan IIA elicits a strong vasodilatory effect in rat and porcine coronary arterioles through the BK_CA_ channel and increased NO and cytochrome P450 metabolites [102, 103]. SalB also can relax the rat coronary artery by inhibiting calcium channels in vascular smooth muscle cells [104]. A systematic review of 60 eligible randomized controlled trials indicates that the Danshen dripping pill, in which SM is the main component, is more effective than isosorbide dinitrate in treating angina pectoris [105].

#### 3.6.2. Myocardial Infarction (MI)

Tan IIA prevents platelet activation by inhibiting the mitogen-activated protein kinase (MAPK) pathway, such as Erk-2 phosphorylation [106]. After MI, reperfusion of ischemic tissue provides oxygen and substrates that are necessary for tissue recovery. However, reperfusion may also induce I/R injury, including excessive production of ROS, enhanced biosynthesis of adhesion molecules, activation of leukocytes, and involvement of cytokines and other inflammatory mediators that cause target and remote organ damage [107]. Both the hydrophilic and lipophilic constituents of SM appear to improve the I/R-induced vascular damage multifactorially and synergistically [108]. The protective function of Tan IIA on myocardial I/R injury may be through inhibiting ROS production and attenuating the expression of high mobility group box B1 protein that results in the activation of proinflammatory pathways [109]. Tan IIA can also reduce monocyte chemoattractant protein-1 expression and macrophage infiltration.

The expression of transforming growth factor-*β*1 in cardiac fibroblasts is inhibited by Tan IIA via the NF-*κ*B signaling pathway [110]. The water-soluble fraction of an SM root extract possesses antioxidant activity, and the hydrophilic components of SM, including protocatechuic aldehyde and Sal B, inhibit the TNF-*α*-induced expression of ICAM-1 and VCAM-1 and the NF-*κ*B and activator protein-1 DNA binding activities in human umbilical vein endothelial cells [111].

Danshensu, the major water-soluble component of SM, protects isolated heart tissue against I/R injury through activation of Akt/ERK1/2/Nrf2 signaling [53]. Recent research shows that Sal A has antiapoptotic effects via activating ERK1/2 and downregulating c-Jun N-terminal kinase (JNK), with increased Bcl-2 and reduced Bax protein expression [112, 113]. A combination of SalB and ginsenoside Rg1 increases the viability of cardiac myocytes and reduces infarct size, thereby improving the functional parameters of the heart against I/R injury in rats [114]. The injection of SM containing water-soluble components such as Sal A shows a cardioprotective effect after infarction by inhibiting L-type Ca^2+^ channels and decreasing the contractility of adult rat cardiac myocytes [115]. Moreover, even the polysaccharide from SM possesses cardiac protective properties [116].

#### 3.6.3. Cardiac Remodeling

Cardiac remodeling is an important aspect of the progression to heart failure observed after MI [117]. Patients with reverse remodeling during treatment have better outcomes and lower mortality than those without such remodeling [118]. Tanshinone VI protects the myocardium against I/R injury and attenuates the progression of myocardial remodeling in vitro [119]. Tan IIA attenuates the expression of angiotensin II-induced collagen type I, ROS formation, and the proliferation of cardiac fibroblasts [120, 121]. Recent research also demonstrates that Tan IIA inhibits extracellular matrix remodeling induced by angiotensin II in human cardiac fibroblasts through inhibition of Smad signaling and MMP-9 expression via nuclear localization of NF-*κ*B [122]. Salvianolic acids, including SalA and Sal B, suppress ROS at the early stage of acute MI and then inhibit the subsequent transcription and posttranslational activation of MMP-9 [123, 124]. Sal B functions as a competitive inhibitor of MMP-9 and inhibits the migration, proliferation, collagen synthesis, and cytokine secretion of cardiac fibroblasts [125, 126].

### 3.7. Stroke

Stroke is the second leading global cause of death behind heart disease [3]. A nationwide population-based study surveyed the usage of traditional Chinese medicine for stroke patients in Taiwan. This study revealed that about 15% of stroke patients used traditional Chinese medicine and that SM was the most used single herb [127]. Disruption of the blood-brain barrier (BBB), inflammatory processes, and nerve cell apoptosis occur after stroke. Tan IIA decreases BBB permeability and suppresses the expression of ICAM-1 and MMP-9 significantly to reduce the infarct area [128]. Another study found that the protective effect of Tan IIA on I/R-induced nerve cells apoptosis involves suppression of excess activation of glial cells, inhibiting the activities of caspase-3 and caspase-8, central regulators of apoptosis [129].

Tan IIA protects the integrity of the BBB through the increased expression of critical endothelial tight junction proteins [130]. Tan IIA is neuroprotective against ischemic stroke, but its short half-life and poor permeability across the BBB limits its effectiveness. There are reports demonstrating that albumin-conjugated PEGylated Tan IIA possesses better brain delivery efficacy and displays remarkable neuroprotective effects through modulation of the MAPK signal pathways and inflammatory cascades [131, 132]. SalB exhibits its neuroprotective effect through antioxidant and antiapoptotic activities by reducing the Bax/Bcl-2 ratio in hippocampal CA1 neurons in mice with I/R injury [133]. The expression of silent information regulator 1, a nicotinamide adenine dinucleotide-dependent deacetylase, is also upregulated by Sal B yielding an antiapoptotic effect after ischemia [134].

A novel derivative of Sal B exhibits a neuroprotective effect against cerebral ischemic injury through angiogenesis and nerve function recovery via the JAK2/STAT3 and VEGF/Flk-1 pathways [135]. Danshensu also possesses a neuroprotective effect against I/R injury by inhibiting apoptosis through activating the phosphoinositide 3-kinase (PI3K)/Akt signaling pathway [136]. Rehabilitation can facilitate some recovery of neurological function after a stroke with evidence of neurogenesis [137]. Sal B promotes neural stem/progenitor cell self-renewal and proliferation through the PI3K/Akt signaling pathway, resulting in improved cognitive function after stroke in rats [138]. These results suggest that SM may act as a potential drug in the treatment of brain injury or neurodegenerative diseases.

### 3.8. Future Prospects

Figures 1 and 2 illustrate the chemical structure and effects of SM on ROS.  Table 1 lists the main antiapoptotic and anti-inflammatory mechanisms of SM. In addition, there are several clinical trials related to SM underway in the United States, including two phase III clinical trials. One is the “Phase III Trial of Dantonic® (T89) Capsule to Prevent and Treat Stable Angina.” The other trial is the “Phase III Study of Compound Danshen Dripping Pills to Treat Diabetic Retinopathy.” Both trials focus on oxidative stress and CVDs. The results will provide important new information about the clinical utility of SM.

The tanshinones are functionally active components in SM. However, they are poorly water-soluble with a low dissolution rate that results in low oral bioavailability. Many studies have focused on improving the drug delivery systems for tanshinones including the use of liposomes [139], nanoparticles [131, 140], and solid dispersions [141]. Further research in this area is needed to assure optimal delivery of SM products.

## 4. Conclusion

SM exhibits antioxidant, antiapoptotic, and anti-inflammatory effects. SM reduces ROS production through inhibiting oxidases, reducing the production of superoxide, inhibiting oxidative modification of LDLs, and ameliorating mitochondrial oxidative stress. It also increases the activities of catalase, MnSOD, glutathione peroxidase, and coupled eNOS. Moreover, in coronary artery disease and stroke, SM not only reduces the impact of I/R injury but also prevents cardiac fibrosis after MI, preserves cardiac function in coronary disease, and maintains the integrity of the BBB, thereby promoting neural stem/progenitor cell self-renewal and proliferation following a stroke. Therefore, SM can be an effective agent for the prevention and treatment of CVDs. However, in accordance with in vitro and in vivo laboratory evidence, well-designed clinical studies are necessary to confirm the efficacy of SM in the treatment of CVDs.

## Figures and Tables

**Table 1:** The main antiapoptotic and anti-inflammatory mechanisms of SM.

	Mechanism	References
*Antiapoptosis*		
Salvianolic acid A	MAPK signaling pathway ↑ERK1/2; ↓JNK with ↓Bax/Bcl-2	[112, 113]
Salvianolic acid B	↑SIRT1; ↓Ac-FOXO1 with ↓Bax/Bcl-2	[134]
Magnesium tanshinoate B	↓JNK, ↓cytochrome c release, ↓caspase-3	[93]
Danshensu	↑PI3K/Akt signal pathway; ↑p-GSK-3*β* levels	[136]
Tanshinone IIA	↓Bax; ↓caspase-3, and ↓Bax/Bcl-2 ratio	[37]
*Anti-inflammation*		
Danshen root	↓IL 8; ↓IL-6 and ↓TNF*α*	[75]
Salvianolic acid B	↑SIRT1; ↓TNF-*α*; ↓IL-1*β*	[134]
Protocatechuic aldehyde	↓NF-*κ*B; ↓AP-1; ↓VCAM-1; ↓ICAM-1	[111]
Tanshinone IIA	↓HMGB1; ↓NF-*κ*B; ↓MCP-1; ↓TNF-*α*; ↓TGF-*β*1; ↓CX3CL1; ↓ICAM- 1; ↓VCAM-1	[90, 109, 110]
Albumin-conjugated PEGylated Tan IIA	↓p38 MAPK; ↓ERK1/2; ↓JNK; iNOS; ↓MPO; ↓TNF-*α*; ↓IL-1*β*; ↓IL-6; ↓TNF-*α*; ↓IL-8; ↓GFAP; ↓MMP-9; ↓COX-2; ↑PPAR*γ*; ↑IL-10; ↑TGF-*β*1	[131, 132]

↑ means upregulation; ↓ means downregulation.

COX-2, cyclooxygenase-2; MAPK, mitogen-activated protein kinase; ERK, extracellular signal-regulated kinase; JNK, c-Jun N-terminal kinase; IL, interleukin; GFAP, glial fibrillary acidic protein; GSK-3*β*, glycogen synthase kinase-3*β*; interleukin; MPO, myeloperoxidase; MCP-1, monocyte chemoattractant protein-1; TNF-*α*, tumor necrosis factor-alpha; TGF-*β*1, transforming growth factor-*β*1; SIRT1, silent information regulator 1; PI3K, phosphoinositide 3-kinase; VCAM-1, vascular cell adhesion molecule; ICAM-1, intercellular adhesion molecule; MMP-9, matrix metalloproteinase-9; PPAR*γ*, peroxisome proliferator activated receptor *γ*.
